# Long-Term Aerobic Exercise Improves Vascular Function Into Old Age: A Systematic Review, Meta-Analysis and Meta Regression of Observational and Interventional Studies

**DOI:** 10.3389/fphys.2019.00031

**Published:** 2019-02-26

**Authors:** Amy Campbell, Fergal Grace, Louise Ritchie, Alexander Beaumont, Nicholas Sculthorpe

**Affiliations:** ^1^School of Health and Life Sciences, Institute of Clinical Exercise and Health Sciences, University of the West of Scotland, Hamilton, United Kingdom; ^2^Faculty of Health, School of Health Science & Psychology, Federation University Australia, Ballarat, VIC, Australia; ^3^School of Sport, York St John University, York, United Kingdom

**Keywords:** vascular aging, vascular function, flow mediated dilation, healthy older adults, exercise

## Abstract

There is an emerging body of literature relating to the effectiveness of frequent aerobic exercise as a prophylactic for age-associated dysfunction of large arteries, yet systematic evaluation and precise estimate of this effect is unknown. We conducted a systematic review and meta-analysis of controlled studies examining flow mediated dilatation (FMD) of athletic older persons and otherwise healthy sedentary counterparts to (i) compare FMD as a determinant of endothelial function between athletes and sedentary individuals and, (ii) summarize the effect of exercise training on FMD in studies of sedentary aging persons. Studies were identified from systematic search of major electronic databases from inception to January 2018. Study quality was assessed before conducting a random effects meta-analysis to calculate a pooled ES (mean difference) with 95% CI's. Thirteen studies [4 interventional (*n* = 125); 10 cross-sectional [including one study from the interventional analysis; (*n* = 485)] with age ranges from 62 to 75 years underwent quantitative pooling of data. The majority of study participants were male. Older athletes had more favorable FMD compared with sedentary controls (2.1%; CI: 1.4, 2.8%; *P* < 0.001). There was no significant improvement in the vascular function of sedentary cohorts following a period of exercise training (0.7%; CI: −0.675, 2.09%; *P* = 0.316). However, there was a significant increase in baseline diameter from pre to post intervention (0.1 mm; CI: 0.07, 0.13 mm; *P* < 0.001). In addition, there was no significant difference in endothelial independent vasodilation between the trained and sedentary older adults (1.57%; CI: −0.13, 3.27%; *P* = 0.07), or from pre to post exercise intervention (1.48%; CI: −1.34, 4.3%; *P* = 0.3). In conclusion, long-term aerobic exercise appears to attenuate the decline in endothelial vascular function, a benefit which is maintained during chronological aging. However, currently there is not enough evidence to suggest that exercise interventions improve vascular function in previously sedentary healthy older adults.

## Introduction

Impaired vascular function as a result of aging occurs due to the coalition of environment, oxidative stress and inflammation (Donato et al., 2007; Seals et al., 2011). These factors result in reduced nitric oxide (NO) bioavailability, causing a failure of the vasculature to dilate in response to increases in shear stress during hyperaemia (Taddei et al., 2000, 2001; Virdis et al., 2010). Furthermore, vascular structure is also compromised with age as wall stiffness increases, reducing flexibility. Therefore, vascular dysfunction promotes cardiovascular disease (CVD) risk and contributes both to a reduction in health span and overall life expectancy (Roger et al., 2012). Given this premise there is an increasingly important but unmet need for interventions which aim to reduce inflammation and oxidative stress, while developing an environment conducive to vascular function (Seals et al., 2009).

Modifiable lifestyle factors, such as increased physical activity (PA) and/or exercise have been advocated to reduce vascular impairment and restore NO dependent vasodilatation, even in apparently healthy older cohorts (Taddei et al., 2000; Grace et al., 2015). Multiple lines of evidence, including both human and pre-clinical models demonstrate that those individuals who are regularly active enjoy superior vascular function, with lower levels of systemic inflammation and oxidative stress (Eskurza et al., 2004; Lesniewski et al., 2013; Seals, 2014; Grace et al., 2015). Despite this more than 1 in 4 of all adults, (The World Health Organization, 2017) and 85–90% of older adults in developed countries fail to meet the PA guidelines to maintain cardiovascular health (Sparling et al., 2015). This represents a contemporary challenge for researchers and healthcare providers to provide evidence-based strategies to improve engagement with PA, and to improve vascular function in older adults as a primary therapeutic target (The World Health Organization, 2010).

Vascular function, or specifically endothelial function, is commonly assessed non-invasively using the flow mediated dilation (FMD) technique. As cardiovascular events can be independently predicted by endothelial compliance, FMD has emerged as a conventional method to determine vascular function (Inaba et al., 2010). Although assessment of whole vascular function includes measures of arterial stiffness, FMD is specific to measuring endothelial function whereby endothelial NO contributes to the vasodilation of vessels after a temporary occlusion of blood flow (Green et al., 2014). However, there are few systematic interrogations of literature examining vascular function of healthy older adults, and those that have been performed, while well-executed, have key limitations. For example, Ashor et al. (2015) and Early et al. (2017) reported that exercise training improved vascular function in healthy and diseased cohorts, but in both cases, data pooling mixed young and old participants, preventing direct assessment of the effect of exercise on vascular function exclusively in older individuals. Moreover, since disease may superimpose additional vascular dysfunction on top of aging alone, it is unclear whether exercise improves vascular function due to direct effects of disease, on age *per se*, or a combination of the two. A further review by Montero et al. (2014) identified that exercise trained adults had displayed superior vascular function compared to their untrained counterparts. However, this review compared trained and untrained adults and did not address the effects of training programmes on vascular function in older adults. Moreover, their inclusion criteria encompassed studies of both middle and old age cohorts. Given that the beneficial effects of exercise may reduce with increasing age, it is difficult to interpret the results of Montero et al. (2014) in an exclusively older population.

Consequently, no meta-analysis has assessed the degree to which older (>60 years) trained individuals may have greater indices of vascular function than their untrained counterparts. Equally, there are no meta-analyses assessing the effectiveness of exercise or PA interventions in improving vascular function in similarly aged, but otherwise healthy adults. Unpicking the relationships between vascular function, aging, and exercise is necessary to enable evidence-based proposals to support health in old age. Therefore, given these gaps in the literature, the aim of this systematic review and meta-analysis was to address the following questions:

- Do longer-term trained older persons have more favorable vascular function, as determined by FMD, than age matched sedentary controls?
- Do short-term exercise training interventions improve vascular function in previously sedentary but healthy older individuals?

## Methods

The current systematic review and Meta-analysis was conducted in accordance with the 2009 Preferred Reporting Items for Systematic Reviews and Meta-Analyses (PRISMA) checklist, and the 2000 Meta-analysis of Observational Studies in Epidemiology checklist (Stroup et al., 2000; Moher et al., 2009).

### Search Strategy

An electronic database search was conducted to identify relevant exercise studies using FMD to determine vascular function of older healthy exercising and sedentary adults. PubMed/MEDLINE (abstract/title), Web of Science (title only) and ScienceDirect (abstract/title/keywords) online databases were searched, and all studies from inception to the date searched (January 2018) were included. The search string included (brachial artery flow mediated dila^*^ OR vasodilation OR vascular function OR vascular reactivity OR vascular health OR endothelial function OR brachial artery) AND (exercise OR train^*^ OR physical activity OR untrain^*^ OR fitness OR program^*^). Filters were applied to ensure that only records in English with human participants were included in the search results. Reference lists from eligible articles were reviewed to search for additional relevant studies which may not have been present during the database search, before being subsequently screened for potential inclusion into the meta-analysis.

### Inclusion and Exclusion Criteria

The inclusion criteria consisted of: (1) non-pharmacological studies of male and female human participants, (2) aged 60 years and over, and (3) employing either cross-sectional, cohort, or randomized control trial (RCT) study designs. (4) Cross sectional design studies had to have an aged matched control group, while in the aerobic exercise intervention, studies both pre and post intervention (cohort) or RCT designs were included. (5) Studies had to include physically healthy cohorts comprising of sedentary individuals for intervention studies, or regular exercisers and sedentary individuals for cross-sectional studies. (6) Vascular function was determined using endothelium dependent FMD of the brachial artery (BA) using valid ultrasonic techniques and occlusion of the lower arm. (7) Studies must have also been published in English language literature. FMD was used as the main measure of vascular function as it is the most widely used non-invasive assessment of endothelial function, and thus gives an accurate representation of endothelial health. As FMD can predict vascular events within asymptomatic persons, it can identify impairments in vascular function within healthy older adults. There was no limitation imposed on the method of subsequent analysis, thus studies using either fixed post-deflation time points or continuous edge detection methods were included. Other measures of vascular function such as pulse wave velocity (PWV) were not included within the meta-analysis as we were specifically interested at measuring endothelial function, rather than arterial stiffness. However, since arterial diameter has been suggested as a potential confounder when assessing FMD (Atkinson and Batterham, 2015) we included analysis of this structural measure. There were also no limitations regarding the length, duration, or intensity of exercise interventions.

Studies were excluded if they (1) used pharmacological stimulus, (2) assessed a single acute exercise bout, (3) assessed resistance interventions only, or (4) assessed vascular function using a method other than FMD. In addition, (5) studies which occluded the upper arm during the FMD protocol were also excluded as this can cause a greater vasodilatory response after ischemia, possibly from mechanisms other than NO (Berry et al., 2000). As the current study aimed to assess the function of the vascular endothelium as a measure of vascular function, only lower arm occlusion was included as the post occlusion vasodilation is mainly NO mediated, and more representative of endothelium function (Doshi et al., 2001; Green et al., 2011). For example, there is evidence that increases in arterial diameter as a result of hyperaemic shear are abolished in the presence of a selective blockage of NO production within lower arm occlusion, but not upper arm occlusion (Doshi et al., 2001). We therefore excluded studies which occluded the upper arm during the FMD protocol as this method causes a greater ischemic response which may not be exclusively due to NO, and thus endothelial function. Furthermore, including lower arm occlusion also helped to standardize the FMD protocol between the included studies.

### Study Selection

The literature search and selection of studies was performed by authors AC and AB. Following an initial screen of titles and abstracts (AC), full scrutiny of potentially eligible studies were independently screened by AC and AB using the specific inclusion criteria. NS arbitrated any disagreements in study inclusion.

### Data Extraction

Data from the final list of eligible studies were extracted and entered into a spreadsheet (Microsoft Excel 2010). Extracted data included the following for all participant groups in each study: (1) participant ages, (2) participant activity status, (3) participant maximum oxygen uptake ($\dot{\text{V}}$O_2max_), (4) sample size, (5) study type, (6) intervention type, frequency, duration and intensity (for interventional studies), (7) relative BA FMD percentage change (ΔFMD%), (8) BA baseline diameter (mm, when reported), (9) endothelial independent vasodilation (EIDV) (%, when measured), (10) supervised and non-supervised interventions, (11) shear rate/stress (when measured), and (12) details of FMD protocols. When studies reported both cross-sectional and interventional data from their analysis, each were screened individually to determine their eligibility. Given the suggestions of morphological adaptations in response to training (Green et al., 2012), we also sought to examine structural changes. Arterial diameter was extracted from studies to determine whether structural adaptations also occurred as a result of exercise. As arterial hyperaemic response is influenced by baseline diameter, extracting arterial diameter may help to understand why some studies show an increase in FMD or not.

ΔFMD% data were extracted as the main outcome variable. If not reported, ΔFMD% was calculated as: [(*post occlusive peak BA diameter* − *baseline BA diameter*)/ (*baseline BA diameter*)^*^100], where post-occlusive diameter was the peak artery diameter which occurred following cuff deflation, and baseline was the diameter determined at rest. All data were entered as mean ± standard deviation (SD). When studies reported standard error of the mean (SEM), conversion to SD was performed using the equation $SD=SEM^{*}\sqrt{\;}\left(N\right)$, where N was the number of participants. Authors of several eligible studies were contacted by email when data were not available from the text, figures, or tables. Where authors failed to respond, mean and standard deviation were extracted from graphs using the calibrated measuring function within the software “ImageJ” (Image Processing and Analysis in Java, Maryland, USA) (Abramoff et al., 2004).

### Study Quality Assessment

Appraisal of study quality was undertaken using assessment tools established by the National Heart, Lung and Blood Institute (NHLBI, Bethesta, MD). Individual quality assessment tools specific to the RCT, (Quality Assessment of Controlled Intervention Studies, 2014) cohort, (Quality Assessment Tool for Before-After (Pre-Post) Studies With No Control Group, 2014) and cross-sectional study designs (Quality Assessment of Controlled Intervention Studies, 2014) were used and subsequently classified as good, fair, or poor.

### Statistical Analysis

All study data were analyzed using the Comprehensive Meta-Analysis software (Biostat: V 2.2.064, Englewood, NJ, USA). Data were entered in accordance with the research questions: (1) ΔFMD% of sedentary participants compared with those who were long-term trained, and (2) pre and post Δ FMD% of previously sedentary participants who had completed an exercise intervention.

The meta-analysis calculated the mean difference (MD) of BA ΔFMD%, BA diameter (mm) and BA EIDV (%), between the long-term trained vs. sedentary participants (question 1) and pre to post intervention in sedentary participants (question 2). A meta-analysis comparing the ΔFMD% of supervised and non-supervised interventions was also included within the analysis. Pooled data were analyzed using a random effects model, and differences in means in a positive direction represented an increase in FMD, baseline diameter and EIDV in favor of exercise, whereas a negative direction indicated a decrease. Between study heterogeneity was calculated for each study question, and reported as Cochran's *Q* and *I*^2^, with variability of measurement characterized as low, medium, or high as 25, 50, and 75%, respectively (Higgins et al., 2003). A method of moments mixed effects meta-regression was conducted to determine if age significantly moderated the effect on ΔFMD%. Publication bias was assessed by Egger's regression. All data are presented as mean ± SD, and a *P*-value of ≤0.05 identified statistical significance.

## Results

### Study Selection

**Figure 1:**
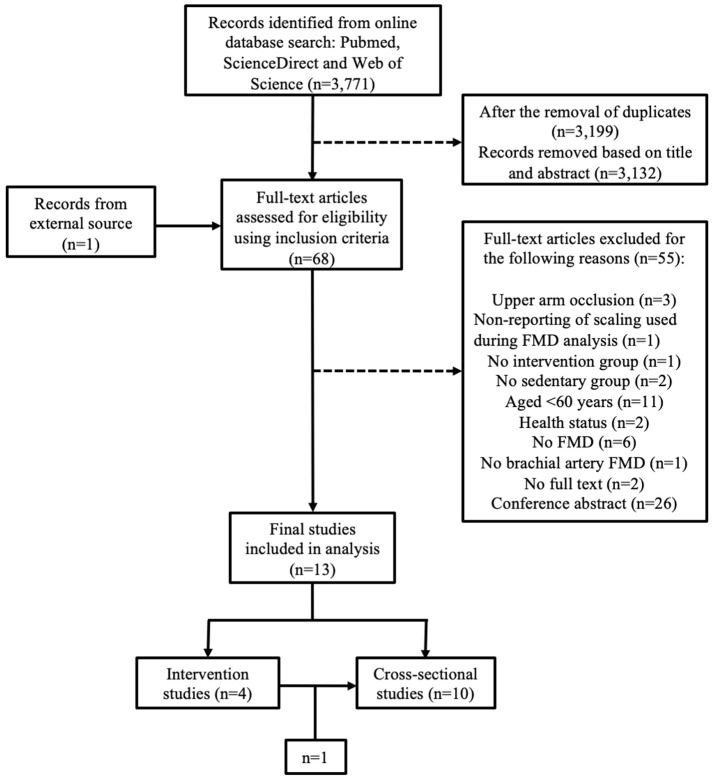
PRISMA flow chart of study selection from the original search on Pubmed, Web of Science, and ScienceDirect.

Following the literature search from all three databases, 3,199 records were identified after the removal of duplicates. Based on title and abstract, 3,132 records were excluded, primarily due to the inclusion of participant morbidity. With the inclusion of 1 study from an external source following reference list examination, full texts of the remaining 68 articles were screened in accordance with the study inclusion and exclusion criteria. Fifty-five studies were excluded for the following reasons: non-reporting of scaling used in FMD analysis (*n* = 1); occlusion of the upper arm during the FMD protocol (*n* = 3); no intervention group (*n* = 1); no sedentary group (*n* = 2); participants were under 60 years of age (*n* = 11); participants were unhealthy (*n* = 2); FMD was not used (*n* = 6); BA FMD was not performed (*n* = 1); conference abstract only (*n* = 26); and no full text available (*n* = 2). Consequently, 13 studies were deemed eligible for analysis. One study consisted of both an intervention and a cross-sectional design and analysis, however only the cross-sectional section met all inclusion criteria (Pierce et al., 2011b). Additionally, one of the interventional studies contained both baseline and post intervention data and was therefore included in both comparisons (Grace et al., 2015). As a result the overall analysis contained 10 cross-sectional (Jensen-Urstad et al., 1999; Eskurza et al., 2004, 2005; Franzoni et al., 2005; Galetta et al., 2006; Walker et al., 2009; Pierce et al., 2011a,b; DeVan et al., 2013; Grace et al., 2015) and 4 interventional studies (Thijssen et al., 2007; Klonizakis et al., 2014; Suboc et al., 2014; Grace et al., 2015) (Figure 1). The included studies were believed to be mainly of “good” quality as measured using the National Heart, Lung and Blood Institute study quality assessment criteria (**Tables 3**–**5**).

The cross-sectional and interventional analyses were found to have moderate and low heterogeneity (*I*^2^ = 63.6%; *P* = 0.003; and *I*^2^ = 47.4, respectively). To compensate for the heterogeneity identified, the meta-analysis was conducted using random effect models, as recommended by the Cochrane guidelines (Higgins and Green, 2011).

### Question 1: Cross-Sectional Study Analysis

The 10 cross-sectional studies included 485 participants (210 long-term trained and 275 sedentary) (Jensen-Urstad et al., 1999; Eskurza et al., 2004, 2005; Franzoni et al., 2005; Galetta et al., 2006; Walker et al., 2009; Pierce et al., 2011a,b; DeVan et al., 2013; Grace et al., 2015). Ages ranged from 62 to 75 years in the sedentary participants (mean of 65 years) and from 61 to 75 years in the long-term trained participants (mean of 65 years). Cohort sizes ranged from *n* = 9 to *n* = 65 participants in the long-term trained and *n* = 9 to *n* = 102 in the sedentary groups. Seven of the long-term trained groups and 5 of the sedentary groups contained <20 participants (Jensen-Urstad et al., 1999; Eskurza et al., 2005; Franzoni et al., 2005; Walker et al., 2009; Pierce et al., 2011a; Grace et al., 2015). Two of the 10 studies contained both male and female participants (Pierce et al., 2011b; DeVan et al., 2013). Studies with both males and females were included as it has previously been identified that post-menopausal females display a similar BA FMD compared to males of the same age (Jensen-Urstad and Johansson, 2001). Additionally, as only one study presented male and female data individually, results for each sex were analyzed together. Long-termed trained participants consisted of endurance runners, swimmers, and cyclists who trained at least three times per week and with regular exercise participation between 2 and 37 years (Table 1).

**Table 1:** Cross-sectional studies characteristics.

**Study**	**Training status**	***N***	**Age (years)**	**% Male**	**Physical activity levels**	**$\dot{\text{V}}$O_**2max**_ (ml.kg.min^**−1**^)**	**FMD % change**	**Overall findings**	**Study Quality**
DeVan et al., 2013	Trained	23	62 ± 4	91	>45 min day^−1^, ≥5 days week^−1^ for previous 2 years	($\dot{\text{V}}$O_2max_): 42 ± 9.6	6.4 ± 1.7	↑ Trained	Fair
	Sedentary	35	62 ± 5	86	<30 min day^−1^, ≤2 days per week for previous 2 years.	($\dot{\text{V}}$O_2max_): 31 ± 5.9	5.3 ± 2.2		
Eskurza et al., 2005	Trained	12	66 ± 4	100	>3 sessions week^−1^ of vigorous endurance exercise >2 years	41.3 ± 4.2	5.9 ± 1.7	↑ Trained	Good
	Sedentary	9	62 ± 6	100	No regular PA >2 years.	29.1 ± 6.3	3.9 ± 2.1		
Franzoni et al., 2005	Trained	16	64 ± 6	100	$\dot{\text{V}}$O_2max_ >50 ml.kg.min^−1^. Vigorous endurance exercise >5 times week^−1^ and participate in national and international road-running races. Training for 37 ± 5 years.	54.7 ± 3.7	5.3 ± 3.2	↑ Trained	Good
	Sedentary	16	64 ± 4	100	$\dot{\text{V}}$O_2max_ <45 ml.kg.min^−1^	28 ± 5.9	2.3 ± 1		
Galetta et al., 2006	Trained	30	65 ± 5	100	$\dot{\text{V}}$O_2max_ >40 ml.kg.min^−1^ Competitive endurance runners since 40 years. 1–2 h day^−1^ for 5 days (3 days long distance running and 2 days walk-weight training) or 5–10 km weekly or 20 km once every 2 weeks.	45.7 ± 3.7	6.2 ± 2	↑ Trained	Good
	Sedentary	28	66 ± 6	100	$\dot{\text{V}}$O_2max_ <35 ml.kg.min^−1^ and no regular exercise	28 ± 5.9	2.4 ± 1.5		
Grace et al., 2015	Trained	17	61 ± 5	100	Life-long exercisers and completed on average 280 min exercise training week^−1^. Most participants were actively competing in endurance sports.	39.2 ± 5.6	5.4 ± 1.4	↑ Trained	Good
	Sedentary	22	63 ± 5	100	No formal exercise programme for ≥30 years.	27.2 ± 5.2	3.4 ± 1.5		
Jensen-Urstad et al., 1999	Trained	9	75 ± 3	100	Participants had been and were still among the best in their respective age groups in running since ages of 15–25. Between 3–7 h strenuous exercise week^−1^.	41 ± 7	4.8 ± 5	↑ Trained	Good
	Sedentary	11	75 ± 2	100	Sedentary or moderately active.	27 ± 5	1.1 ± 2.1		
Pierce et al., 2011a	Trained	13	62 ± 7	100	Vigorous aerobic exercise (competitive running, cycling and triathlons) ≥ 5 days week^−1^ for ≥ 45 min day^−1^ >5 years.	42 ± 3.6	6.3 ± 1.8	↑ Trained	Good
	Sedentary	28	63 ± 5	100	No regular aerobic exercise (<30 min day^−1^, <2 days week^−1^, ≥2 years).	29 ± 5.3	4.9 ± 2.1		
Pierce et al., 2011b	Trained	65	62 ± 6	69	Vigorous aerobic exercise (competitive running, cycling and triathlons) >5 days week^−1^ for >45 min day^−1^ >5 years.	41.5 ± 7.7	6.1 ± 2.9	↑ Trained	Good
	Sedentary	102	62 ± 10	59	No regular aerobic exercise (<30 min day^−1^, <2 days week^−1^, >2 years).	27.4 ± 6.6	4.8 ± 2.3		
Walker et al., 2009	Trained	16	66 ± 4	100	>3 sessions week^−1^ vigorous aerobic endurance exercise.	42.8 ± 5.2	6.2 ± 2.6	→	Good
	Sedentary	15	66 ± 4	100	No regular exercise for 2 years	29.9 ± 4.7	4.8 ± 1.6		
Eskurza et al., 2004	Trained	9	64 ± 6	100	>3 sessions week^−1^ vigorous aerobic endurance exercise for ≥ 2 years	40 ± 6	7 ± 1.8	↑ Trained	Good
	Sedentary	9	64 ± 6	100	Sedentary (No regular PA) for ≥ 2 years	32 ± 3	4.6 ± 0.6		

*Study characteristics of cross-sectional studies within question 1 [↑ identifies that a significant increase in FMD% change occurred (P ≤ 0.05) and → identifies no change (P > 0.05)]. N, number of participants; % Male, percentage of male participants: $\dot{V}$O_2max_, maximum aerobic capacity; FMD% change, flow mediated dilation percentage change; PA, physical activity). Values are displayed as mean ± SD*.

Studies described cuff occlusion pressures to range from 40 mmHg above systolic blood pressure to 300 mmHg and remained inflated between 4 and 5 min. ΔFMD% was analyzed from all 10 cross-sectional studies and ranged from 4.8 ± 5 to 7 ± 1.8% in the long-term trained participants and 1.1 ± 2.1 to 5.3 ± 2.2% in sedentary participants. ΔFMD% values normalized for shear stress were reported in one study (Eskurza et al., 2005), and for hyperaemic shear in one other (Eskurza et al., 2004). All 10 cross-sectional studies reported mean baseline BA diameter data, whereas only 9 studies reported EIDV data.

**Figure 2:**
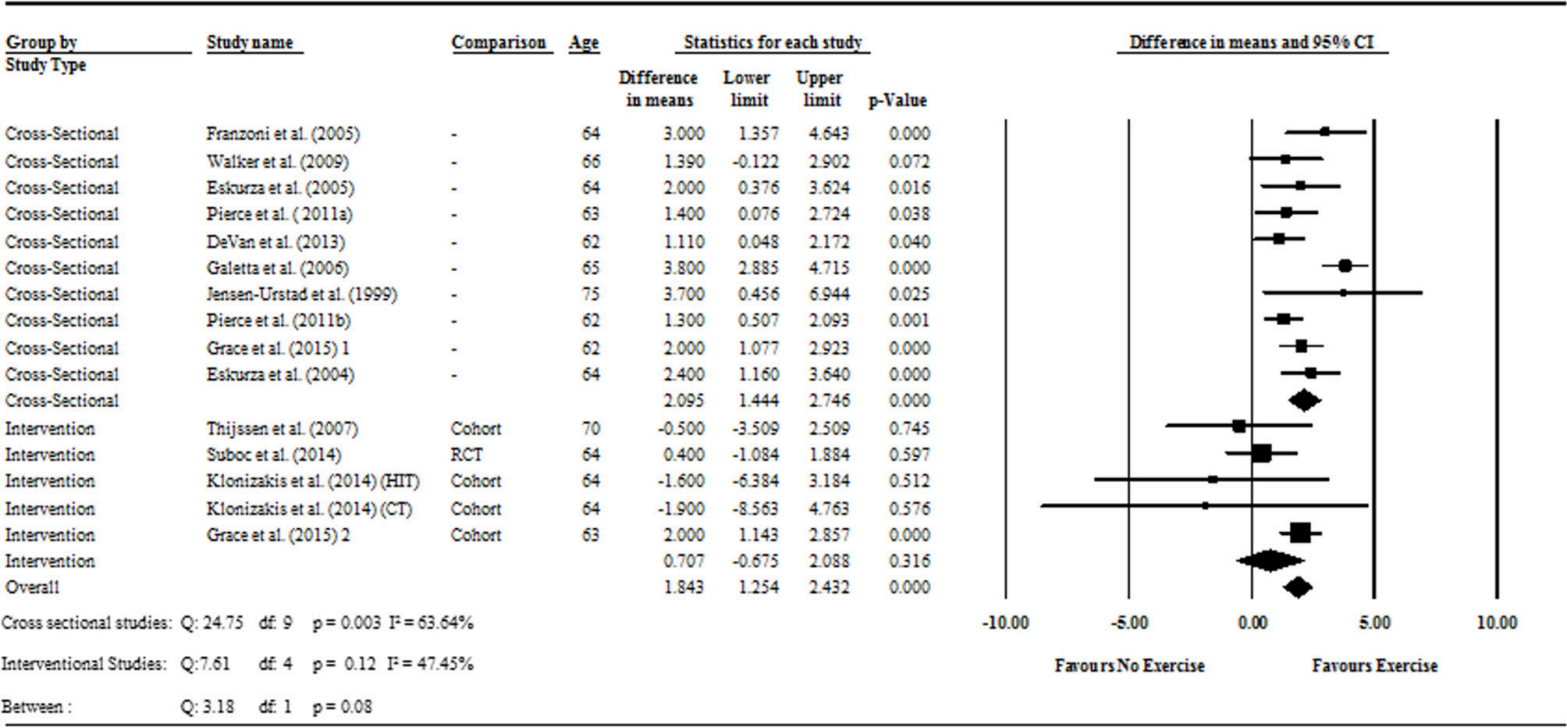
Forest plot of the meta-analysis with mean differences of FMD percentage change between trained vs. sedentary healthy older adults (question 1), and FMD percentage change pre to post exercise intervention in previously sedentary healthy older adults (question 2). Outcomes of questions 1, 2, and the moderator analysis are also presented.

**Figure 3:**
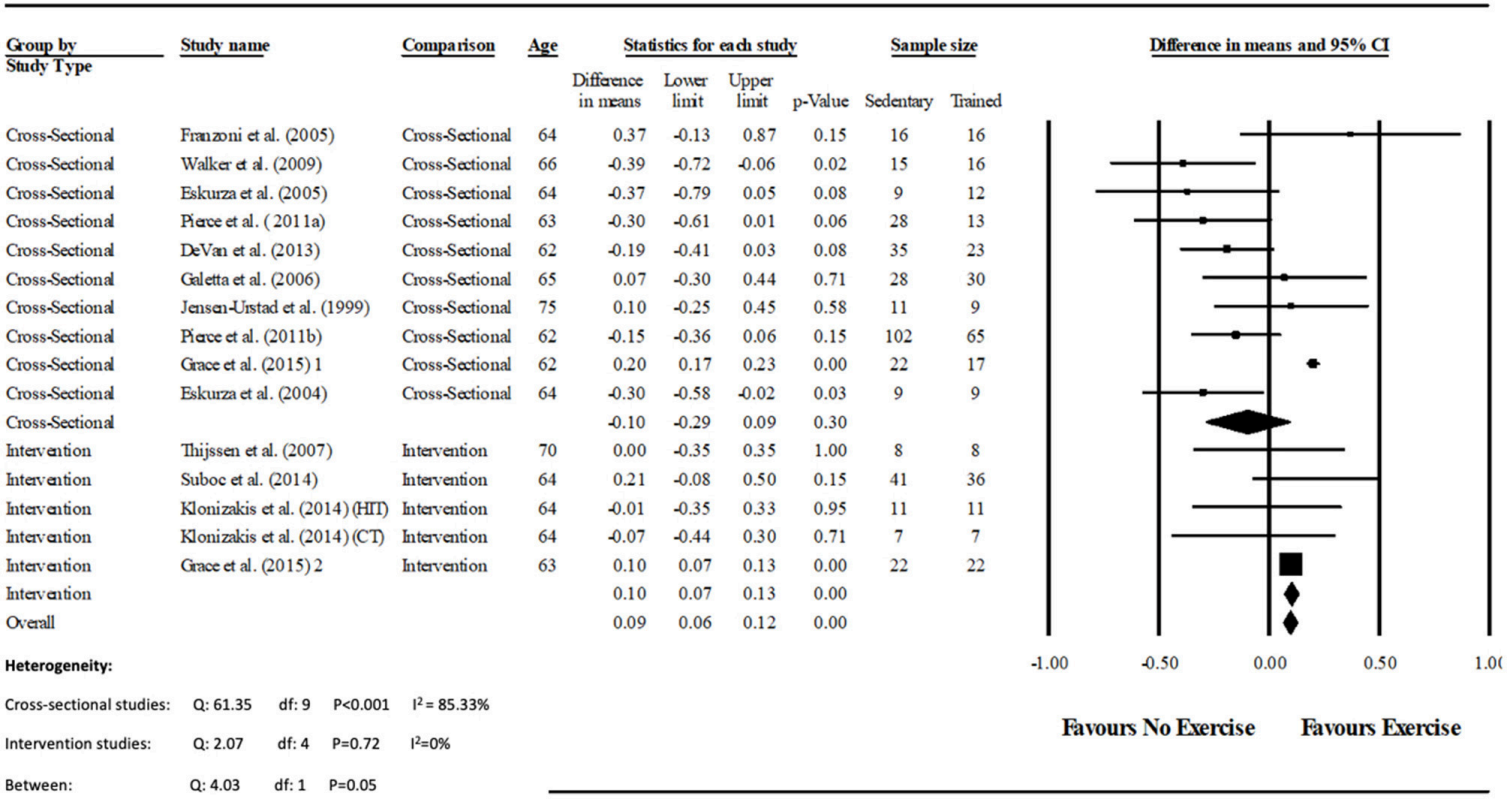
Forest plot of the meta-analysis of brachial artery baseline diameter (mm) between trained vs. sedentary healthy older adults, and pre to post exercise intervention in previously sedentary healthy older adults. The heterogeneity and moderator analysis are also presented.

**Figure 4:**
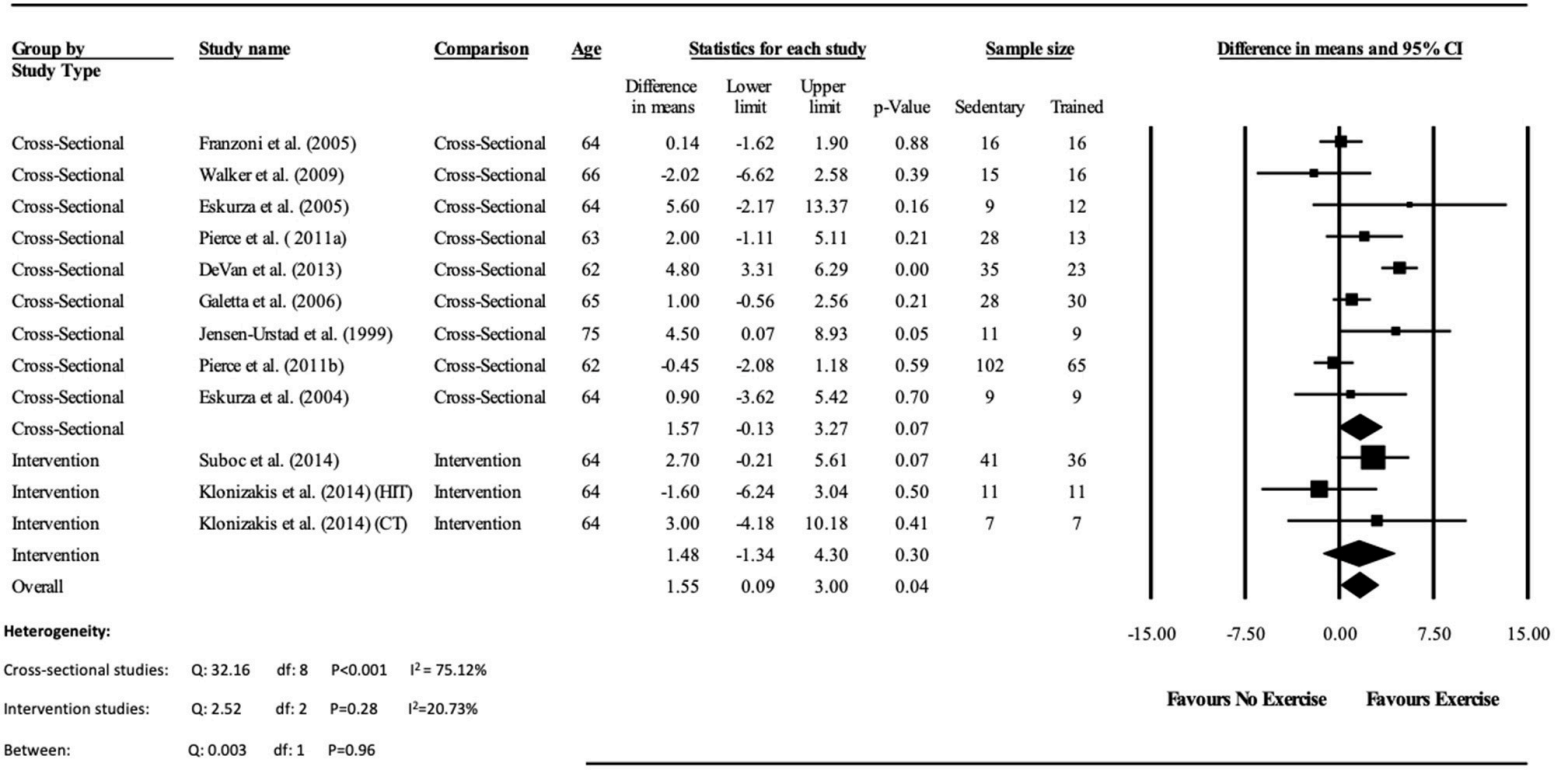
Forest plot of the meta-analysis of endothelial independent vasodilation percentage change (EIDV%) between trained vs. sedentary healthy older adults, and pre to post exercise intervention in previously sedentary healthy older adults. The heterogeneity and moderator analysis are also presented.

Data pooling from the meta-analysis indicated that ΔFMD% was significantly greater in long-term trained vs. sedentary older adults (MD: 2.1, 95% CI: 1.4, 2.8%; *P* < 0.001; Figure 2). Moderate heterogeneity was observed between the 10 cross-sectional studies (*I*^2^ = 63.6%; *P* = 0.003). Egger's regression determined that there was a low risk of publication bias (*P* = 0.7). The meta-regression found no significant effect of age on ΔFMD% (*P* = 0.08; Table 6). Data pooling identified that EIDV was not significantly different between the trained and untrained participants (MD: 1.57; 95% CI: −0.132, 3.274%; *P* = 0.07; Figure 4), and baseline diameter of the BA was also similar between the two groups (MD: −0.1 mm; 95% CI: −0.09 mm, 0.29 mm; *P* = 0.30; Figure 3).

### Question 2: Intervention Study Analysis

The 4 intervention studies consisted of 3 cohort designs (Thijssen et al., 2007; Klonizakis et al., 2014; Grace et al., 2015) and one RCT (Suboc et al., 2014). Included in the 4 studies were 125 sedentary participants. Sample sizes ranged from 8 to 77 participants, where two of the studies contained <20 participants (Thijssen et al., 2007; Klonizakis et al., 2014). The ages of participants ranged from 62 ± 7 to 70 ± 1 years, with a mean age of 65 years. One of the studies contained both male and female participants (Suboc et al., 2014), and one study recruited only postmenopausal females (Klonizakis et al., 2014). Again, as the study containing both males and females did not present their results separately, data for each sex was analyzed together (Table 2).

**Table 2:** Interventional study characteristics.

**Study**	**Study design**	**N**	**Age (years)**	**% Male**	**Exercise intervention**				**Study duration**	**$\dot{\text{V}}$O_**2max**_ (ml.kg.min^**−1**^)**	**FMD % change**	**Overall findings**	**Study Quality**
					**Exercise type**	**Intensity**	**Session duration**	**Frequency**					
Thijssen et al., 2007	Cohort	8	70 ± 1	100	Cycling training on an ergometer	65% HRR and gradually increasing by 5% until 85%	20 min	3 days week^−1^	8 weeks	Pre: 30.8 ± 4.8	Pre: 6.9 ± 3.4	→ FMD%	Good
										Post: 33.3 ± 5.5	Post: 6.4 ± 2.7		
Suboc et al., 2014	RCT	77	PED: 64 ± 7	PED: 61	PED (*n* = 36) walking CON (*n* = 41)	Increase PA by 10% weekly above baseline to reach an average of 10,000 steps day^−1^	–	Daily	12 weeks	–	Post: CON:6.3 ± 2.7	→ FMD%	Good
			CON: 62 ± 7	CON: 76							HIT:6.7 ± 3.9		
Klonizakis et al., 2014	Cohort	18	HIT: 64 ± 7	0	HIT (*n* = 11): cycling intervals on ergometer	HIT: 100% PP and light active recovery intervals at 30 W	HIIT: 10 × 1 min intervals with 1 min recovery between each	3 times week^−1^	2 weeks	HIT; Pre: 20.4 ± 3.4 Post: 22.6 ± 3.1	HIT; Pre:8.1 ± 7.2 Post:6.5 ± 3.7	→ FMD%	Good
			CT: 64 ± 4		CT (*n* = 7): continuous cycling	CT: 65% PP	CT: 40 min			CT; Pre: 25 ± 7.4 Post: 26.7 ± 5.4	CT; Pre: 8.9 ± 7.9 Post: 7 ± 4.3	→ FMD%	
Grace et al., 2015	Cohort	22	63 ± 5.2	100	Progressive conditioning exercise: ACSM guidelines	Conditioning exercise ACSM guidelines (Chodzko-Zajko et al., 2009) and 50% PP HIIT sprints	Conditioning exercise: 150 min week^−1^ ≥30 min day^−1^ (ACSM guidelines Chodzko-Zajko et al., 2009)	Conditioning: ≥5 days week^−1^ (ACSM guidelines Chodzko-Zajko et al., 2009)	Conditioning: 6 weeks	Pre: 27.2 ± 5.2	Pre: 3.4 ± 1.5	↑ FMD%	Good
					HIIT: sprints on cycle ergometer.		HIIT: 6 × 30 s sprints with 3 min break between each	HIIT: once every 5 days	HIIT: 6 weeks	Post: 32.2 ± 5.6	Post: 5.4 ± 1.4		

*Study characteristics of interventional studies within question 2. ↑ identifies a significant increase in FMD% change (P ≤ 0.05) and → identifies no significant change (P > 0.05). N, number of participants; % Male, percentage of male participants; $\dot{V}$O_2max_, maximum aerobic capacity; FMD% change, flow mediated dilation percentage change; PA, physical activity; HRR, heart rate reserve; WR_max_, maximum work rate; PP, peak power; HIT, high intensity training; CT, continuous training; ACSM, American College of Sports Medicine; HIIT, high intensity interval training; PED, pedometer; CON, control. Values are displayed as mean ± SD*.

Cuff inflation during the FMD protocol ranged from 50 mmHg above systolic blood pressure to approximately 220 mmHg for a 5 min period. The duration of study interventions lasted between approximately 9 to 84 sessions from 2 to 12 weeks, with 3 of the studies including exercise interventions of a moderate to high intensity. One of the cohort studies included two separate interventions—one consisting of high intensity, and the other a moderate intensity intervention (Klonizakis et al., 2014). Frequency of interventions ranged from once every 5 days to 7 days per week and lasting between 20 min to 1 h per day.

For the cohort studies ΔFMD% was calculated from baseline and post measures, while post intervention and control values were analyzed in the RCT study. ΔFMD% ranged from 3.4 ± 1.5 to 8.9 ± 7.9% pre-intervention to 5.4 ± 1.4 and 7 ± 4.3% post intervention. All four of the studies reported baseline BA diameter data, whilst only two studies reported data for EIDV.

**Figure 5:**
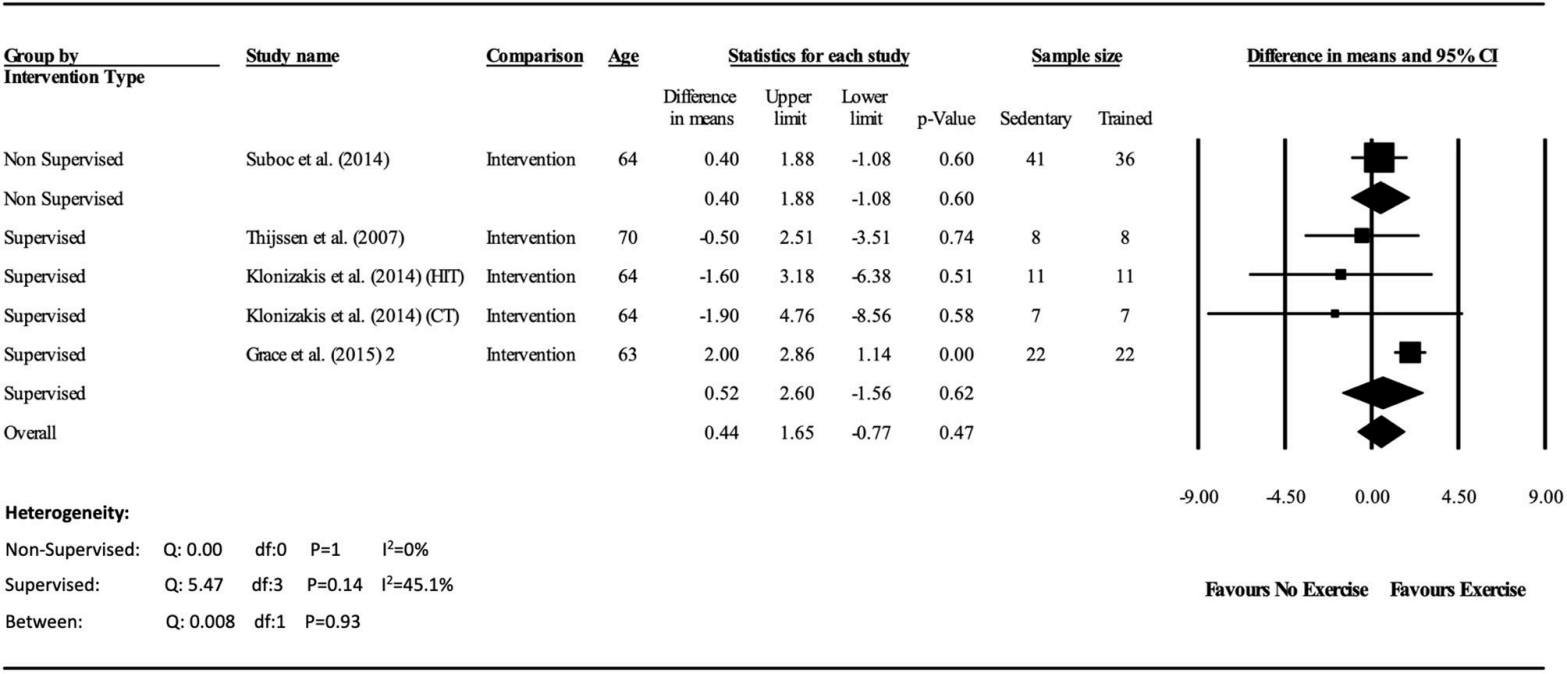
Forest plot of the meta-analysis of brachial artery FMD percentage change within supervised and non-supervised exercise interventions. Heterogeneity and moderator analysis are also presented.

The meta-analysis suggests that there was no significant improvement in ΔFMD% after the exercise interventions in previously sedentary older adults (MD: 0.707, 95% CI: −0.68, 2.1%; *P* = 0.316; Figure 2). Heterogeneity of the 5 intervention studies were calculated as low (*I*^2^ = 47.4%, *P* = 0.107, and Egger's regression determined that some publication bias may be present (*P* = 0.047). The meta-analysis identified that there was no increase in EIDV from pre to post intervention (MD: 1.48; 95% CI: −1.3, 4.3%; *P* = 0.303; Figure 4), however there was a significant increase in BA baseline diameter post intervention (MD: 0.1 mm; 95% CI: 0.07 mm, 0.13 mm *P* < 0.001; Figure 3). Finally, ΔFMD% was not affected by whether the interventions were supervised or non-supervised (heterogeneity *p* = 0.927; Figure 5).

## Discussion

This systematic review and meta-analysis set out to determine the effects of short and long-term exercise training on vascular function and has 2 main findings. First, pooled data from cross-sectional studies demonstrate that long-term trained healthy older adults have superior vascular function compared with their sedentary but otherwise healthy counterparts; and second that FMD may not improve in sedentary individuals who undertake shorter-term aerobic exercise interventions although there may be an increase in arterial diameter. These data are the first pooled synthesis of controlled observational and interventional studies using healthy older cohorts. The current meta-analysis also allows some comparison between observational and interventional studies since we used the same inclusion and exclusion criteria for studies in both comparisons. Moreover, since all participants were apparently healthy and not taking any medication, the results may provide some insight into the effect of short and long-term training on aging *per se* rather than on aging in combination with comorbidities. The current study therefore differs from previous meta-analyses which combined healthy and diseased participants, or mixed middle aged and older participants together in their analysis (Montero et al., 2014; Ashor et al., 2015; Early et al., 2017). Therefore, to our knowledge the current study is the first meta-analysis to identify vascular function exclusively in healthy adults aged 60 years and over who are either endurance trained or untrained, or who have completed an endurance intervention having previously been sedentary.

### Question 1—Do Long-Term Trained Older Individuals Demonstrate Superior Vascular Function, as Determined by FMD, Than Age Matched Sedentary Controls?

As outlined above, the meta-analysis indicated that endurance exercise training is associated with improved vascular function, despite considerable differences in study designs. Indeed, within these 10 studies there was a wide range in prior exercise experience, with trained cohorts ranging from a minimum of 2 years training through to life-long exercisers and which may have contributed to the heterogeneity in this comparison.

Nevertheless, the data indicates that exercise provides a protective mechanism in long-term trained participants, who experience a slower deterioration of vascular function, compared with sedentary but otherwise healthy older adults. While the protective mechanism of exercise remains to be fully elucidated it is widely believed that the hyperaemic effects of exercise, and the repeated exposure of the endothelium to bouts of increased shear stress, act to reduce the deleterious effects inflammation and oxidative stress (Tinken et al., 2010). The combination of these effects enhances the bioavailability of NO, increasing vasodilation during the hyperaemic response following an ischaemic stimulus (Taddei et al., 2000). From the data presented it appears that the vasculature of healthy older long-term trained adults may be exposed to these physiological mechanisms at a level which preserves vascular function relative to their sedentary counterparts.

Previous work has suggested that trained individuals have shown to exhibit wider peripheral artery diameters when compared to untrained individuals. Often referred to as the athletes artery, it is thought to represent arterial remodeling in response to repeated bouts of shear stress which occur during exercise. It is hypothesized that a widened vessel requires less vasodilation during periods of reactive hyperaemia, resulting in reduced dilatation during FMD (Green et al., 2012, 2013). However, the analysis of long-term trained athletes, in whom any such adaptation may be greatest fails to support this concept since arterial diameters of the trained individuals did not differ significantly from the sedentary cohort. Although allometric scaling of the data would have been beneficial, a lack of anthropometric data within the studies meant that scaling was not possible. Nevertheless, the present meta-analysis identified that the trained group had a greater vascular function compared to the untrained group, whilst both displaying a similar baseline artery diameter (Figure 3). These findings agree with a meta-analysis by Montero et al. (2014) who identified that masters athletes BA diameters were similar to untrained controls, whilst FMD was also significantly greater.

The meta-regression aimed to identify if increasing age reduced the improvement in FMD seen in trained individuals compared to sedentary controls. In this case the lack of a significant association indicated that the difference between the two cohorts was not reduced with advancing age Therefore, the present findings underline the notion that exercise can support vascular function, well into the eight decade (Grace et al., 2015). It is also notable, that most (Walker et al., 2009; Pierce et al., 2011a; DeVan et al., 2013), but not all (Franzoni et al., 2005), previous work has demonstrated that the vascular function of healthy young untrained adults is greater than older trained adults. These data suggest that, even in well-trained individuals, there may some unavoidable decrement in vascular function with age. In addition, the range in mean ages of the included observational studies was relatively narrow (62–75 years); future research should attempt to determine if improvements in vascular function is maintained in those who participate in long-term endurance exercise.

### Question 2—Do Exercise Training Interventions Improve Vascular Function in Previously Sedentary Healthy Older Persons?

The results of observational studies support the use of exercise to ameliorate the age related decline in vascular function. However, as is the case in all observational studies, the inability to directly link the main outcome (i.e., FMD) to the exposure of interest (i.e., exercise training) limits the confidence that may be placed in conclusions from such studies (Higgins and Green, 2011). In an attempt to overcome this, the present review further assessed the effectiveness of prospective aerobic intervention studies with otherwise sedentary participants.

Data pooling indicated that the short term training programmes within the included studies (2–12 weeks) did not significantly improve vascular function. This finding is at odds with the results of the cross-sectional assessment of endurance trained participants and controls reported above. However, the training interventions were associated with a significant increase in diameter of the BA at baseline (i.e., immediately prior to cuff inflation; Figure 3). Since included studies where not mechanistic in nature, assessing the lack of effect is speculative. For example, it may be that in this age group the recovery of vascular function is slow and vascular remodeling (i.e., “the athletes artery”) becomes a primary method of adaptation (Green et al., 2012). Similarly, it may be that since most studies used cycling exercise, early adaptation is focussed on the most active vascular beds of the lower limbs, and assessment of brachial arterial function is insufficiently sensitive (Thijssen et al., 2007).

Alternatively, there may not be any vascular dysfunction, and the lack of effect on FMD is a result of the increased baseline diameter which aids total blood flow. Indeed increased shear stress, as a result of an exercise has been suggested to cause systemic arterial remodeling (Maiorana et al., 2011). It is noteworthy that long-term training improved FMD without morphological changes (rationale 1), while short term training increased baseline diameter without improving FMD. Given the interventions were between 2 and 12 weeks, and that vascular remodeling may occur within that time frame (Tinken et al., 2008), it is possible that this is responsible for increased baseline diameter within short term interventions. However, the majority of the interventional studies only measured vascular function before and after the exercise intervention. The “pre-post” nature of FMD assessments means that it is possible that improvements were missed by the time post measures where performed. Indeed, future work should assess the presence (or otherwise) of changes in vascular function throughout the time course of training interventions. However, it is also worth noting that there was no improvement in vascular function after 2 weeks of either continuous or high intensity training within the study by Klonizakis et al. (2014), suggesting that relatively fast improvements in vascular function may not always occur.

It is also worth noting several limitations of the literature pertaining to interventions in this age group. There were a relatively small number of interventional studies which met the inclusion criteria, meaning that again there was a relatively limited age range of 62–70 years. Moreover although the studies included within the meta-analysis were endurance based, the 4 included studies consisted of low, moderate and high exercise intensities, and ranged between 2 and 12 weeks, all of which may have contributed to the moderate heterogeneity of the pooled data. Future analyses may benefit from analyzing intensity zones as these may affect vascular function differently (Ashor et al., 2015; Ramos et al., 2015; Early et al., 2017), and the greatest effect in the available literature was observed following high intensity interval training (Grace et al., 2015). However, due to the limited number of studies included within the current analysis, it was not possible to categorize studies based on their intensities.

However, despite the lack of improvements in vascular function following exercise interventions, there are a number of other physiological advantages of exercise such as increased muscle strength and power (Reid and Fielding, 2012), as well as a reduction in other risk factors involved in aging (Seals, 2014; Barnes, 2015). It therefore seems prudent to advise older adults who begin exercising to remain active indefinitely in order to enjoy other health benefits associated with PA and exercise; not least as detraining may reverse potential improvements in various physiological factors (Pullin et al., 2004).

Additionally, the meta-analysis identified that EIDV did not change significantly in either the trained vs. untrained participants in the cross-sectional analysis, or pre vs. post training within the interventional analysis, however combined pooling suggested a small effect. As EIDV is commonly used as a control test to assess whether improvements in FMD are mainly NO mediated, these data suggest that improvements in trained participants FMD may be due to exercise induced improvements of the vascular endothelium, rather than alterations in vascular smooth muscle within the tunica media (Maruhashi et al., 2013). Conversely, the previously sedentary participants who completed an exercise intervention found no overall improvements in either endothelial function, or vascular smooth muscle cell function.

### Study Quality

The quality of most studies was determined as “good” (see Tables 3–5); however, some limitations were noted. Firstly, the majority of both observational and cohort studies recruited only a small number of participants (<20 participants per group) which increases the chances of type 2 error, while also increasing the risk of finding a disproportionately large effect size (Button et al., 2013). Furthermore, the current meta-analysis only identified a single RCT from the literature which met the study's inclusion criteria. Given that RCTs are widely considered the most internally valid design to determine cause and effect (Evans, 2003), future studies should investigate the use of this approach. Furthermore, few studies reported that outcome assessors were blind to the participant's group allocation during assessment of FMD, increasing the risk of performance bias (Higgins and Green, 2011).

**Table 3:** Cross-sectional study quality.

	**Research question clear?**	**Study population clear? (including without date and place)**	**Criteria same within both groups?**	**Sample size calculation?**	**Exposure measures clear, reliable and valid? Implemented consistently?**	**Outcome measures clear, reliable and valid? Implemented consistently?**	**Blinded assessment of FMD?**	**Follow-up after baseline 20% or less?**	**Key potential confounding variables considered?**
DeVan et al., 2013	Y	Y	Y	N	N	N	NR	Y	Y
Eskurza et al., 2005	Y	Y	Y	N	Y	Y	Y	Y	Y
Franzoni et al., 2005	Y	Y	Y	N	Y	Y	NR	Y	Y
Galetta et al., 2006	Y	Y	Y	N	N	Y	NR	Y	Y
Grace et al., 2015	Y	Y	Y	Y	Y	Y	N	Y	Y
Jensen-Urstad et al., 1999	Y	Y	Y	N	N	Y	NR	Y	Y
Pierce et al., 2011a	Y	Y	Y	N	N	Y	Y	Y	Y
Pierce et al., 2011b	Y	Y	Y	N	Y	Y	Y	Y	Y
Walker et al., 2009	Y	Y	Y	Y	Y	Y	NR	Y	Y
Eskurza et al., 2004	Y	Y	Y	N	Y	Y	Y	Y	Y

*Study quality assessment of the cross-sectional studies included in question 1 developed by the National Heart, Lung and Blood Institute (NHLBI) (Quality Assessment Tool for Observational Cohort Cross-Sectional Studies, 2014). Y, yes; N, no; NR, not reported*.

**Table 4:** Cohort study quality.

	**Research question clear?**	**Participant eligibility criteria clear? (without date and names)**	**Were participants representative of general population of interest?**	**Were all eligible participants enrolled?**	**Was sample size sufficient to provide confidence in the findings?**	**Was the intervention clearly described and delivered consistently?**	**Outcome measures clear, reliable and valid? Implemented consistently?**	**Blinded assessment of FMD?**	**Loss to follow-up after baseline <20%**	**Did the statistical method examine changes pre and post intervention?**
Grace et al., 2015	Y	Y	Y	Y	Y	Y	Y	NR	Y	Y
Klonizakis et al., 2014	Y	Y	Y	Y	CD	Y	Y	NR	Y	Y
Thijssen et al., 2007	Y	Y	Y	Y	Y	Y	Y	NR	Y	Y

*Study quality assessment of the cohort studies included in question 2 developed by the National Heart, Lung and Blood Institute (NHLBI) (Quality Assessment Tool for Before-After (Pre-Post) Studies With No Control Group, 2014). Y, yes; N, no; NR, not reported; CD, cannot determine*.

**Table 5:** Randomized control trial study quality.

	**Described as a RCT?**	**Was the method of randomization adequate?**	**Was the treatment allocation concealed?**	**Were the investigators blinded?**	**Were groups similar at baseline on important characteristics that could affect outcomes?**	**Was overall drop-out rate ≤20%**	**Was the differential drop-out rate at endpoint ≤15%?**	**High adherence to intervention protocols?**	**Were other interventions avoided or similar in the groups?**	**Outcome measures clear, reliable and valid? Implemented consistently?**	**Sample size sufficient to detect difference with at least 80% power?**	**Were outcomes reported or subgroups analyzed pre-specified?**	**Were all randomized participants analyzed in the group they were originally assigned?**
Suboc et al., 2014	Y	NR	NR	Y	Y	Y	Y	N	Y	Y	Y	Y	Y

*Study quality assessment of the RCT study included in question 2 developed by the National Heart, Lung and Blood Institute (NHLBI) (Quality Assessment of Controlled Intervention Studies, 2014). RCT, randomized controlled trial; Y, yes; N, no; NR, not reported*.

**Table 6:** Meta-regression analysis.

**Comparison**	**Covariate**	**n**	**q**	**df**	**SE**	**β**	**95% CI**	***P*-value**
Trained vs. sedentary	Age	10	3.17	1	0.12	0.22	0.02, 0.46%	0.08

*Meta-regressions assessing the effect of age on FMD percentage change*.

Moreover, while the included intervention studies performed outcome assessments at the cessation of the training programme, no studies included a sufficient follow-up to allow for determination of the longevity of any beneficial effect. Although FMD is a useful predictor of vascular endothelial function, longer follow-up periods would be useful to identify whether improvements in FMD from exercise translates into a decreased incidence of vascular disease and mortality. Additionally, there is also the need for more comprehensive reporting of participant characteristics, including confirmation of medical history, training status, training frequency and duration (e.g., mins per week), and intensity as many studies lack sufficient detail.

### Strengths and Limitations

This is the first systematic review and meta- analysis to focus on the effects of exercise on vascular function via FMD in healthy older adults using both cross-sectional and interventional studies; however, a number of limitations should be noted. Firstly, although FMD measurement protocols were similar between studies, some minor differences in protocols were evident. Studies measured post occlusion BA diameter for different durations, ranging from 90 s to 10 min after cuff deflation. In addition to differences in methodology, there were also a wide range of baseline ΔFMD% values in intervention studies (3.4 ± 1.5 to 8.9 ± 4.9%). Furthermore, only a small number of studies normalized FMD values for hyperaemic stimulus (Eskurza et al., 2004, 2005). Therefore, the varied protocols and lack of FMD normalization by shear may have all contributed to the differences between the studies. Also, for the purposes of this analysis we defined vascular function as brachial artery FMD response. However, it is plausible that analysis of micro-vascular function, different regions of the vascular system, or analysis of other measures of vascular health (e.g., pulse wave analysis or pulse wave velocity) may provide different results.

Moreover, although the interventional studies were specifically aerobic in nature, the intensities of the exercises differed considerably. For example, Suboc et al. (2014) conducted a walking intervention, which was of relatively low intensity, whereas (Klonizakis et al., 2014) documented a high intensity protocol requiring participants to work at 100% of their peak power output. However, due to the lack of interventional studies meeting the inclusion criteria, all 4 studies had to be analyzed together. A greater number of interventional studies are therefore required within this specific cohort to investigate whether varying exercise intensities would influence vascular improvements differently. Additionally, within the cross-sectional studies the lack of detail, and the use of qualitative descriptors of training intensity in the trained older individuals (e.g., “vigorous exercise”) makes more detailed comparison of the effects of long-term training in this cohort difficult. Furthermore, although there were no differences in ΔFMD% between supervised and non-supervised interventions, only one non-supervised intervention was included in the meta-analysis. Therefore, until further non-supervised interventional protocols meeting the inclusion criteria become available, this result should be interpreted with caution.

All of the studies included within the meta-analysis assessed vascular function of the BA via FMD, despite the interventions and longer-term exercise routines consisting primarily of lower-limb exercise. However, it has previously been identified that cycling can significantly improve vascular function of the non-exercising upper limbs (Birk et al., 2012). Although Birk et al. (2012) assessed only young male participants, their results suggest that lower-limb exercise can cause systemic adaptations to vascular function, which is likely caused by increases in shear stress. Therefore, it seems likely that the improvement in BA vascular function identified within the cross-sectional studies is due to systemic vascular adaptations from many years of lower-limb exercise training.

Additionally, it has been previously shown that vascular function in older females decreases at a faster rate than in males (Celermajer et al., 1994). As the current systematic review and meta-analysis included both male and female participants, it is possible that our results could have differed if males and females were analyzed separately. However, there were too few studies containing female participants to split the analysis by sex. Furthermore, it has been previously found that estrogen may be required to induce the benefits of endurance exercise on vascular function, potentially by increasing NO bioavailability further (Moreau et al., 2013). As the females included in the meta-analysis were post-menopausal and not receiving estrogen supplementation, perhaps intake of the hormone after menopause alongside aerobic exercise may have helped to improve vascular function further. However, as the majority of the cross-sectional participants within the current meta-analysis were male, the results from the cross-sectional analysis may better represent the male population. Future studies may wish, where possible, to report male and female results separately, and to further report the menstrual status of female participants.

## Conclusion

In summary, the current systematic review and meta-analysis identifies that aerobic exercise training during advancing age can maintain healthy vascular function compared with otherwise healthy sedentary peers. These findings emphasize the importance of remaining active throughout the life-span. However, currently there is not enough evidence to suggest that aerobic exercise interventions ranging from 2 to 12 weeks can improve vascular function in previously sedentary older adults. Nonetheless, sedentary older individuals should still be encouraged to become active until more evidence becomes available.

## Author Contributions

The literature search and selection of studies was performed by authors AC and AB. Following an initial screen of titles and abstracts (AC), full scrutiny of potentially eligible studies were independently screened by AC and AB using the specific inclusion criteria. NS arbitrated any disagreements in study inclusion. Study quality assessment was performed by AC. All authors contributed to the development of the final manuscript.

### Conflict of Interest Statement

The authors declare that the research was conducted in the absence of any commercial or financial relationships that could be construed as a potential conflict of interest.
